# Comparison of User Satisfaction and Preference with Inhalant Devices Between a Pressurized Metered-Dose Inhaler and Ellipta in Stable Asthma Patients: A Randomized, Crossover Study

**DOI:** 10.1007/s41030-021-00149-6

**Published:** 2021-03-02

**Authors:** Hiroyuki Ohbayashi, Takamitsu Asano, Sahori Kudo, Mitsue Ariga

**Affiliations:** Department of Allergy and Respiratory Medicine, Tohno Chuo Clinic, Mizunami, Japan

**Keywords:** Adherence, Asthma, Device operability, Inhalant device, User satisfaction

## Abstract

**Introduction:**

Inhalation therapy involves two types of adherence: adherence to the drug and adherence to the procedures for the inhalation device. User satisfaction and preference are key factors for maintaining good adherence of both types, and they should be evaluated based on three conditions being well maintained: asthma control level (ACL), adherence, and adequate device operability during usage duration. We compared user satisfaction and preference between a pressurized metered-dose inhaler (pMDI) and a dry powder inhaler (Ellipta), while maintaining the three conditions during usage in stable asthma patients.

**Methods:**

In this open-label, randomized, two-way crossover study, patients with stable asthma [Asthma Control Questionnaire (ACQ) scores < 0.75] were classified into a 20–64-year age group (G1) and a ≥ 65-year age group (G2) and randomly assigned to either a formoterol/fluticasone combination (FFC) as the pMDI group or a vilanterol/fluticasone combination (VFC) as the Ellipta group. Satisfaction and preference levels were evaluated at week 4. ACL was measured using the ACQ and Japan Asthma Control Survey questionnaires at weeks 0 and 4. Device operability and respiratory resistance were also examined.

**Results:**

Forty-four patients (23 G1, age 45.8 ± 1.9 years; 21 G2, 74.1 ± 1.3 years) were enrolled and maintained good ACL during the study. Adherence to FFC pMDI and VFC Ellipta was > 97% in all groups. Device operability did not differ significantly between FFC pMDI and VFC Ellipta in the G1 (*p* = 0.189) or G2 (*p* = 0.506) group. Overall satisfaction was marginally higher with the FFC pMDI than with the VFC Ellipta in G2 (*p* = 0.012) but non-significantly different in G1 (*p* = 0.733). Factors affecting overall satisfaction in G2 were difference of inhalation device and body mass index. Respiratory resistance did not change significantly over the study in G2.

**Conclusion:**

Based on maintaining good ACL, adherence, and device operability, FFC pMDI showed significantly higher satisfaction and preference levels than VFC Ellipta in elderly persons.

**Trial Registration:**

Japan Registry of Clinical Trials identifier, jRCTs041180001 (registered 21 August 2018).

**Supplementary Information:**

The online version contains supplementary material available at 10.1007/s41030-021-00149-6.

## Key Summary Points

**Table:** 

**Why carry out this study?**
Inhalation therapy involves two types of adherence, adherence to the drug and adherence to the procedures for the inhalation device, and these factors contribute to the clinical effects of inhaled corticosteroid/long-acting beta agonist (ICS/LABA) combination therapy.
User satisfaction and preference are key factors for maintaining good adherence of both types, and they should be evaluated based on three conditions being well maintained: asthma control level, adherence, and adequate device operability during usage duration.
No studies have directly compared different inhalation devices of ICS/LABA products based on these three conditions being well maintained.
**What was learned from the study?**
While maintaining these three conditions, a pressurized metered-dose inhaler showed significantly higher satisfaction and preference levels than a dry powder inhaler in stable elderly asthmatic patients.
There is a diversity of satisfaction and preference among patients, which may change with time based on various factors, such as age. The results obtained in this study may provide useful hints for electing an inhaler device in clinical settings.

## Digital Features

This article is published with digital features, including a summary slide, to facilitate understanding of the article. To view digital features for this article go to https://doi.org/10.6084/m9.figshare.13713187.

## Introduction

A major difference between inhalation therapy and conventional oral medication therapy is that two types of adherence should be kept in mind with inhalation therapy [1]: adherence to the drug and adherence to the procedures for the inhalation device. The success of inhalation therapy stands on these two legs of adherence, and if either is neglected, the basis of the inhalation therapy will not be established. Patient satisfaction and preference for the inhalation therapy are crucial factors in maintaining adherence and are determined by patients’ satisfaction not only with the medication itself, but also with the inhalation device. In actual clinical settings, however, patients are often not satisfied with the inhalation device [2]. One reason for this is that physicians’ main prescribing criteria are often based on pharmacological information, such as the drug’s mechanism of action and clinical evidence. The selection of drug also determines the device to be used, and in some cases, the drug is started without adequately considering the device. Despite the extreme importance of patient satisfaction and preference for the device in maintaining adherence, these components are often not sufficiently reflected in the drug prescription [3]. Continued use without obtaining satisfaction is a factor in the eventual collapse of overall adherence to the inhalation therapy [4].

Moreover, unless the patient uses the device for a certain minimum period and has an opportunity to use another comparable device, the patient will have no basis on which to judge satisfaction or preference with regard to the device used. Different devices also exhibit different patterns of lung deposition, which may significantly affect clinical potency for lung function and individual clinical effects [5, 6]. In this study, patient satisfaction and preference were investigated for two of the latest asthma inhalation therapy drugs in Japan: a combination of vilanterol and fluticasone delivered with an Ellipta dry powder inhaler device (VFC Ellipta), and a combination of formoterol and fluticasone delivered with a pressurized metered-dose inhaler device (FFC pMDI). The components of VFC Ellipta and FFC pMDI were different; however, these devices containing different molecules were evaluated because of their availability in Japan. The VFC Ellipta has great convenience, with inhalation once a day using a simple inhalation technique in which the drug is set in the device by simply opening the cover. The VFC Ellipta shows a higher level of satisfaction than fluticasone propionate/salmeterol Diskus and conventional pMDI preparations [7], and it has been reported that patients have a significantly higher preference for it even when there is no significant difference in respiratory function or on the Asthma Control Test (ACT) [8]. Thus, there seem to be factors affecting patient satisfaction other than the clinical efficacy of the drugs. The FFC pMDI, unlike conventional pMDI devices, was developed so that the spray velocity is gentler for easier breathing coordination, and the use of a spacer/valved holding chamber is typically not necessary. Moreover, with an inhalation support device (Full Push), the cylinder bottom can be pushed with less finger force. With these characteristics, it is possible that patient satisfaction and preference with regard to the FFC pMDI will differ from a reported investigation with conventional pMDI preparations that found they were difficult to use [9].

It has been reported that adherence declines and inhalation technique errors occur frequently, especially in older patients, resulting in poorer asthma control [10]. Thus, we conducted the study to compare user satisfaction and preferences of two devices in an elderly group and a non-elderly group, respectively.

## Methods

The study protocol was approved (Japan Registry of Clinical Trials No. jRCTs041180001, 21/Aug/2018) by the research ethics committee of Fujita Health University Certified Clinical Research Review Board CRB4180003 (Toyoake, Aichi, Japan) before study initiation. The study was carried out in accordance with the principles embodied in the Helsinki Declaration of 1995 (as revised in 2013). All study participants provided informed consent.

### Study Design

**Fig. 1:**
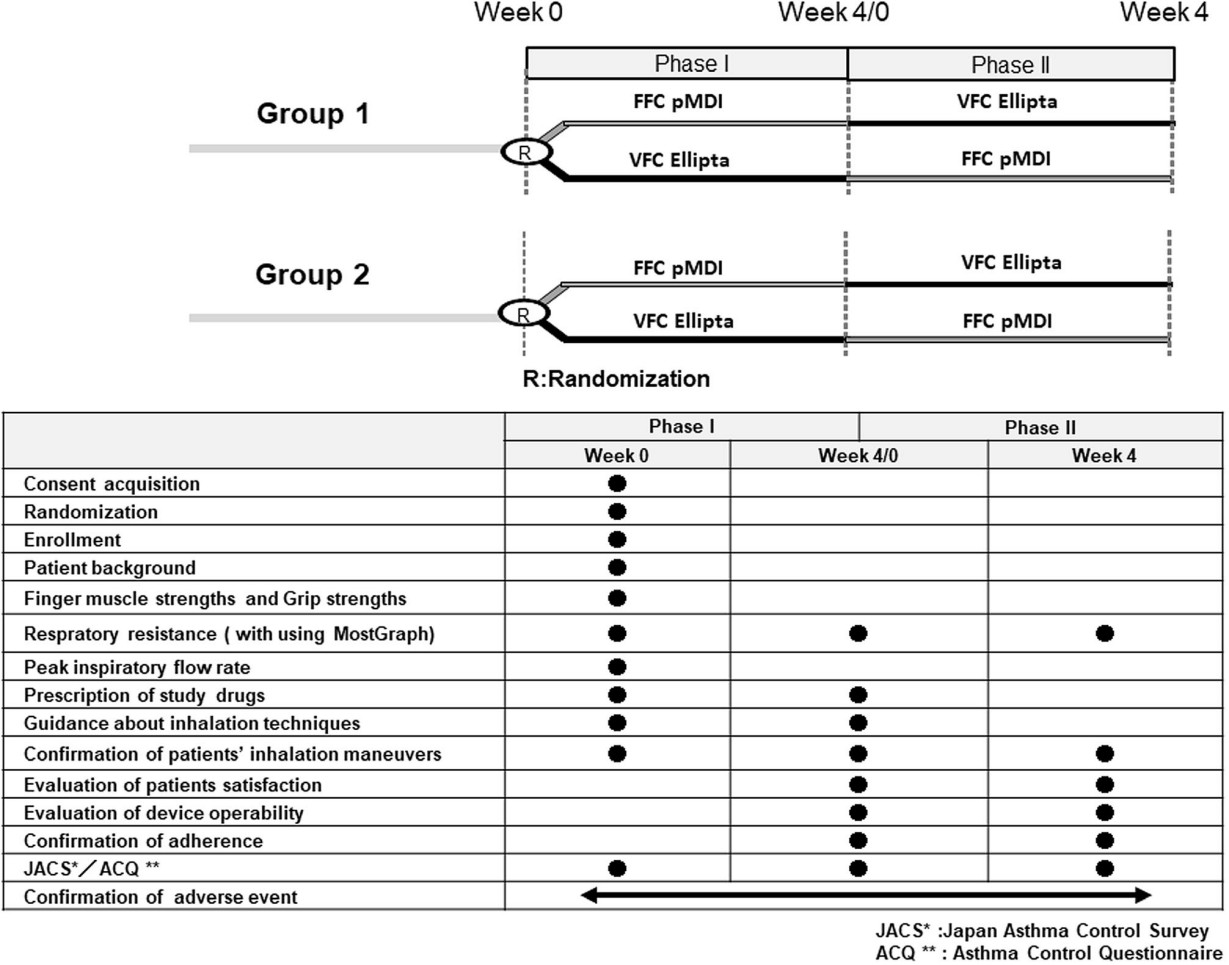
Study design. The primary outcome of this randomized, two-way crossover study is overall degree of satisfaction. Each menu of the evaluation items performed at weeks 0 and 4 is shown below the study design

This clinical study was an open-label, randomized, two-way crossover study involving asthma outpatients who visited Tohno Chuo Clinic (Gifu, Japan) between October and December 2018. As shown in Fig. 1, patients were randomly assigned to either the FFC pMDI or VFC Ellipta groups at a 1:1 ratio with a permutated block method at week 0 and classified by age (20–64 years in group 1, ≥ 65 years in group 2). Outcomes were evaluated at week 4 and crossed over to the other group (Fig. 1).

This clinical study was based on three important preconditions: patients had achieved stable asthma control; they maintained good adherence during the study; and they received inhalation instruction and could perform the appropriate inhalation technique. Patients received specific training in inhalation device procedures from a trained nurse instructor at week 0. The instructor repeated the training to ensure that patients mastered device operability and procedures perfectly using each checklist (Supplement).

### Patient and Public Involvement

There was no patient or public involvement.

### Medications Used

The medications used were as follows: VFC Ellipta, vilanterol 25 µg and fluticasone 200 µg combination, one inhalation once a day (morning at a regular time) (GlaxoSmithKline, Middlesex, UK); and FFC pMDI, formoterol 5 µg and fluticasone 125 µg combination, two inhalations twice a day (morning and evening at regular times) (Kyorin Pharmaceutical Co., Ltd, Tokyo, Japan). The Full Push aid was attached to FFC pMDI in all cases, and a spacer was not used.

### Study Outcomes

The primary study outcome was the overall degree of satisfaction, the answer to Q5 in the patient satisfaction questionnaire (Supplement). Satisfaction level, operability, and adherence were collected at week 4. Patient satisfaction and device operability were measured by questionnaire responses. Inhaler adherence was measured by the dose counters on the inhaler devices to count the number of inhalations during the 4-week study period. The asthma control questionnaire (ACQ) and the Japan Asthma Control Survey (JACS) questionnaire were evaluated at weeks 0 and 4 to confirm persistent good asthma control. The JACS is an original Japanese questionnaire with 15 questions about symptoms, activities, emotions, and treatment with regard to the optimal measurement of asthma control status. The reliability of the JACS questionnaire is similar to that of the ACT [11]. The cut-off values of 8.0 and 4.8 are appropriate for differentiating well-controlled, not well-controlled, and poorly controlled asthma as defined by the criteria in the Japanese Asthma Prevention and Management Guideline 2015 [12].

Respiratory resistance and reactance were measured using forced oscillation technique (FOT) measurements by MostGraph-01 (Chest M.I., Inc., Tokyo, Japan) at weeks 0 and 4. Respiratory resistance at 5 Hz (R5), respiratory resistance at 20 Hz (R20), and R5–R20, as well as low-frequency reactance indices at 5 Hz (X5), resonant frequency (Fres), and low-frequency reactance area (ALX) were measured using the FOT. The patients’ background characteristics were checked at week 0, and finger muscle and grip strengths were determined at week 0.

### Statistical Analysis

**Fig. 2:**
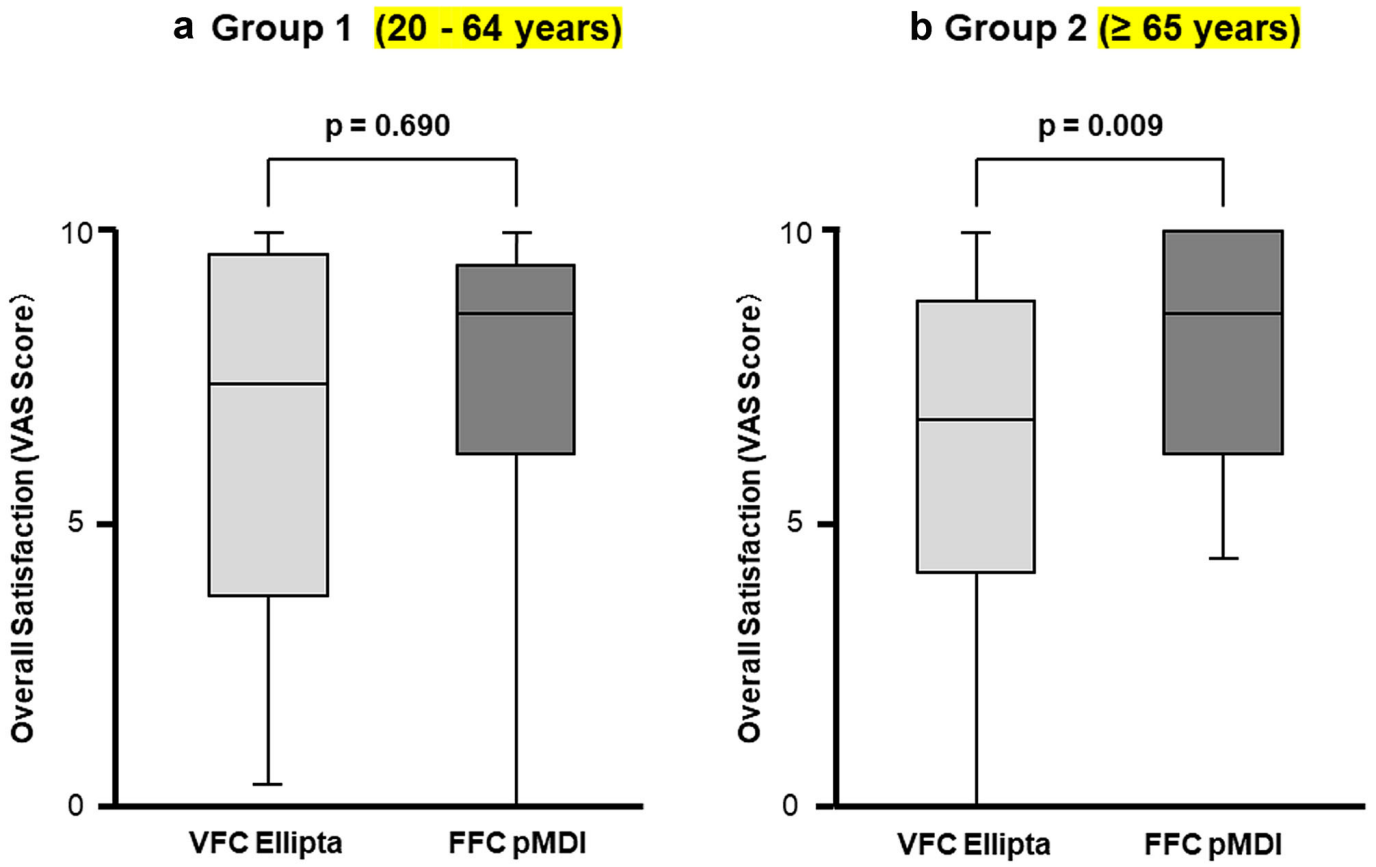
Comparison of overall satisfaction between the inhalation devices in groups 1 and 2. The overall satisfaction with FFC pMDI was significantly higher than with VFC Ellipta in group 2 (*p* = 0.009), but not in group 1 (*p* = 0.690)

**Fig. 3:**
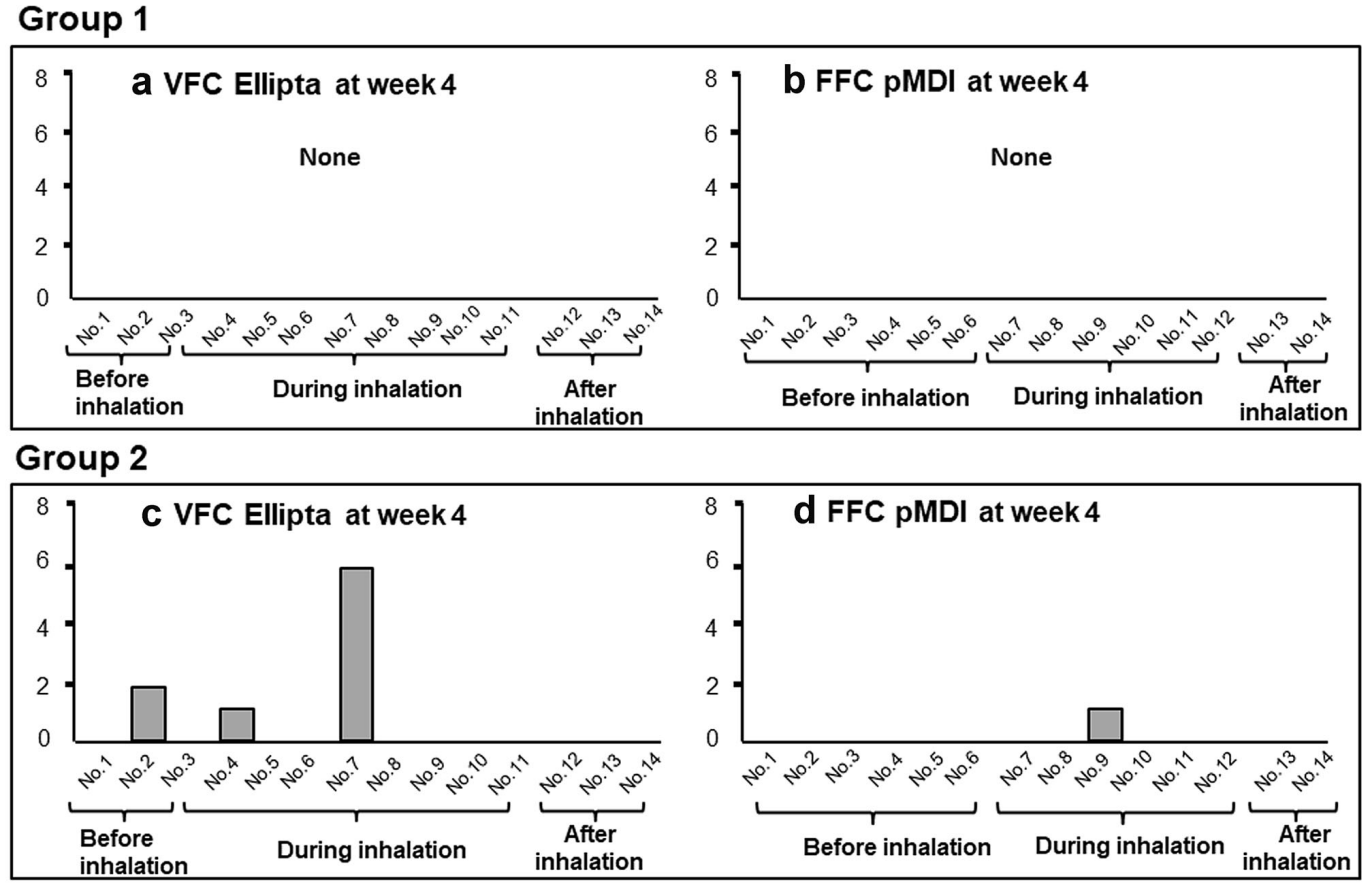
Error counts in group 1 and group 2 at week 4. There were no device handling errors with the VFC Ellipta or FFC pMDI in group 1 at week 4. Handling errors with both devices occurred at week 4 in group 2

The significance level was < 5%. Normal distribution was checked by the Shapiro–Wilk test. Non-parametric data are presented as maximum, first quartile, third quartile, minimum, and median values. We conducted the Wilcoxon rank-sum test for between-group differences or Wilcoxon signed-rank test for within-group differences as non-parametric tests. We conducted Fisher’s exact test for comparison of nominal data in contingency tables. Patient characteristics were compared between groups 1 and 2 with the unpaired Student’s *t* test or Fisher’s exact test (Table 1). Correlations among peak inspiratory flow, finger muscle strength, and grip strength were evaluated with Pearson’s correlation coefficient after checking the normal distribution of data. Comparisons of the results of ACQ scores, JACS scores, respiratory function, patient satisfaction questionnaires, and device handling error counts at week 4 between the FFC pMDI and VFC Ellipta groups in groups 1 and 2 were performed with the Wilcoxon rank-sum test (Tables 2, 3, Figs. 2 and 3). The responses of those who answered questions concerning device operability at week 4 were compared with Fisher’s exact test (Table 5)**.** All statistical analyses were performed using JMP version 14.1.0 for Windows (SAS Institute, Cary, NC, USA).

**Table 1:** Patient baseline characteristics

Characteristic	Group 1 Non-elderly group (*N* = 23)	Group 2 Elderly group (*N* = 21)	*p* value Fisher’s exact test	*p* value Student’s *t* test
Sex (male), *n* (%)	9 (39.1)	5 (23.8)	0.342	–
Age (years), mean (SD)	45.8 (9.3)	74.1 (5.8)	–	< 0.001
BMI (kg/m^2^), mean (SD)	23.0 (4.3)	23.5 (2.9)	–	0.659
Disease type				
Atopic, *n* (%)	12 (52.2)	5 (23.8)	0.069	–
Non-atopic, *n* (%)	11 (47.8)	16 (76.2)		
Severity				
Moderate persistent, *n* (%)	23(100)	21 (100)	1.000	–
Disease duration (months), mean (SD)	27.0 (33.6)	20.9 (23.5)	–	0.494
Treatment step				
Step 3, *n* (%)	23 (100)	21 (100)	1.000	–
Previous medication, *n* (%)			0.767	–
Fluticasone/salmeterol Diskus	10 (43.5)	8 (38.1)		
Budesonide/formoterol Turbuhaler	13 (56.5)	13 (61.9)		
Complications				
Yes: *n* (%)	15 (65.2)	17 (81.0)	0.318	–
Smoking history				
Yes: *n* (%)	7 (30.4)	4 (19.1)	0.494	–
Grip strength (kg), mean (SD)	33.1 (11.0)	21.9 (10.9)	–	0.002
Finger muscle strength (kg), mean (SD)	7.0 (2.7)	5.0 (2.2)	–	0.011
Peak inspiratory flow (L/min), mean (SD)	215.2 (100.3)	147.9 (70.9)	–	0.015
Pulmonary function (pulmonary resistance using MostGraph)				
Mean (SD)				
R5 (cmH_2_O/L/s)	2.3 (0.9)	3.0 (1.3)	0.031	–
R20 (cmH_2_O/L/s)	2.2 (0.6)	2.7 (0.8)	0.020	–
R5–R20 (cmH_2_O/L/s)	0 (0.5)	0.3 (0.6)	0.190	–
X5 (cmH_2_O/L/s)	−0.2 (0.3)	−0.5 (0.5)	0.024	–
Fres (Hz)	7.8 (2.6)	9.7 (3.9)	0.073	–
ALX (cmH_2_O/L/s Hz)	1.1 (1.0)	2.6 (3.6)	0.066	–

**Table 2:** Results of questionnaires concerning asthma control level

Device	Group 1 (DPI:23 pMDI:22)					Group 2 (DPI:21 pMDI:21)				
	Mean at week 0	Mean at week 4	Within-group change	Within-group *p* value*	Between-group *p* value**	Mean at week 0	Mean at week 4	Within-group change	Within-group *p* value*	Between-group *p* value**
ACQ 5										
VFC	0.10	0.10	0.00	0.625	0.698	0.08	0.06	−0.01	0.703	0.717
FFC	0.07	0.03	−0.05	0.120		0.06	0.07	0.01	0.983	
ACQ 6										
VFC	0.08	0.08	0.00	0.612	0.698	0.06	0.05	−0.02	0.307	0.878
FFC	0.06	0.02	−0.04	0.120		0.05	0.06	0.01	0.983	
JACS total score										
VFC	8.63	8.54	−0.09	0.712	0.937	8.72	8.56	−0.16	0.867	0.513
FFC	8.57	8.64	0.07	0.637		8.68	8.71	0.03	0.987	
JACS activity										
VFC	8.58	8.36	−0.22	0.268	0.159	8.48	8.47	−0.01	0.839	0.724
FFC	8.44	8.61	0.18	0.330		8.50	8.57	0.06	0.316	
JACS treatment										
VFC	8.29	8.55	0.26	0.221	0.203	8.88	8.84	−0.04	0.949	0.363
FFC	8.30	8.21	−0.09	0.628		8.83	8.65	−0.18	0.297	
JACS symptoms										
VFC	8.69	8.64	−0.05	0.939	0.770	8.81	8.48	−0.33	0.150	0.129
FFC	8.75	8.67	−0.08	0.910		8.73	8.78	0.06	0.520	
JACS mental										
VFC	8.81	8.67	−0.14	0.902	0.842	8.85	8.60	−0.25	0.270	0.186
FFC	8.68	8.85	0.17	0.897		8.77	8.84	0.07	0.486	

*Within-group differences were analysed by the Wilcoxon signed-rank test

**Between-group differences were analysed by the Wilcoxon rank-sum test

**Table 3:** Results of questionnaire regarding the degree of patient satisfaction

Questionnaire	Device	Group 1			Group 2		
		Median	(25–75%)	*p* value*	Median	(25–75%)	*p* value*
Q1	VFC Ellipta	4.9	2.6–9.7	0.024	6.5	3.2–8.9	0.001
	FFC pMDI	9.9	5.2–10		9.7	7.8–10	
Q2	VFC Ellipta	Data shown in Table 4			Data shown in Table 4		
	FFC pMDI						
Q3	VFC Ellipta	9.6	6.7–10	0.672	8.2	4–9.9	0.099
	FFC pMDI	9.8	7.3–10		9.3	6.6–10	
Q4	VFC Ellipta	6	4.7–9.4	0.785	5.6	2.8–8.4	0.080
	FFC pMDI	7.1	1.9–9.2		7.4	6.1–9	
Q5 (also shown in Fig. 2)	VFC Ellipta	7.4	5–9.8	0.690	6.7	4–9	0.009
	FFC pMDI	8.7	5.4–9.8		8.6	7.1–10	
Average of Q1, Q3, Q4 and Q5	VFC Ellipta	7.0	5.0–9.5	0.364	6.8	4.7–8.8	0.017
	FFC pMDI	8.3	5.6–9.4		8.9	7.3–9.9	

*Wilcoxon rank-sum test

**Table 4:** Detailed answers concerning Q2 in Table 3

Group 1				Group 2			
VFC Ellipta *n* = 23		FFC pMDI *n* = 22		VFC Ellipta *n* = 21		FFC pMDI *n* = 21	
Negative answer reason							
Powdery feeling	7	Taste of inhalation medicine	2	Powdery feeling	5	Able to breathe along with inhalation	2
Powdery feeling and choking	2	Discomfort of month and tongue	2	Powdery feeling and choke	2	Smell	2
Shape of mouthpiece	2	Smell	2	Discomfort of month and tongue	2	Hoarseness	1
Cough	2	Hoarseness	1	Shape of mouthpiece	2	Powdery feeling	1
Discomfort of month and tongue	1	Not easy to use	1	Lever was hard	2		
Lever was difficult to move	1	Able to breathe along with inhalation	1	Change of taste	1		
Trouble for once daily when symptom appeared	1	Possible to forget twice daily	1	Leaking of powder before use	1		
Trouble for once daily when forgetting inhalation	1	Counter	1	Cough	1		
Positive answer reason		Easy to use	1			Easy to use	1

**Table 5:** Answers concerning evaluation of device operability at week 4

Evaluation questionnaire	Group 1			Group 2		
	Proportion of those who answered 4 points (fair) or 5 points (good)			Proportion of those who answered 4 points (fair) or 5 points (good)		
	VFC Ellipta	FFC pMDI	*p* value*	VFC Ellipta	FFC pMDI	*p* value*
Q1	21/23**	20/22	1.000	18/21	16/21	0.697
Q2	16/23	18/22	0.491	17/21	18/21	1.000
Q3	21/23	19/22	0.665	20/21	19/21	1.000
Q4	19/23	16/22	0.491	18/21	18/21	1.000
Q5	21/23	12/22	0.007	15/21	14/21	1.000
Q6	13/23	14/22	0.763	14/21	14/21	1.000
Q7	18/23	16/22	0.738	15/21	14/21	1.000
Q8	19/23	14/22	0.189	13/21	16/21	0.506

*Q1* Are the usage instructions easy to understand? *Q2* Is the inhaler easy to carry? *Q3* Are the preparations for inhaling simple? *Q4* Is it easy to inhale? *Q5* Does the smell bother you? *Q6* Are you conscious of the medication while inhaling? *Q7* Do you feel that you are inhaling correctly? *Q8* What is your overall impression of the inhaler?

*Fisher exact test

**Denominator: total patients including this evaluation. Numerator: patients sum of “fairly good” and “good”

## Results

Twenty-three outpatients with stable moderate asthma in group 1 (age 45.8 ± 9.3 years) and 21 outpatients in group 2 (74.1 ± 5.8 years) were enrolled. Their characteristics are shown in Table 1.

The primary endpoint, i.e. the results of the Q5 questionnaire item, are shown in Fig. 2. Overall satisfaction was significantly higher with FFC pMDI than with VFC Ellipta in group 2 (*p* = 0.009), but not group 1 (*p* = 0.690). There were significant differences in grip strength (kg) (*p* = 0.002), finger muscle strength (kg) (*p* = 0.011), and peak inspiratory flow (L/min) (*p* = 0.015) between groups 1 and 2, respectively. The other factors including body mass index (BMI) (*p* = 0.659) showed no significant differences. There were significant correlations between peak inspiratory flow and grip strength (*r* = 0.784, *p* < 0.001), peak inspiratory flow and finger muscle strength (*r* = 0.313, *p* = 0.015), and finger muscle strength and grip strength (*r* = 0.340, *p* = 0.113) in group 1. In group 2, there were significant correlations between grip strength and body mass index (BMI) (*r* = 0.374, *p* = 0.095), grip strength and finger muscle strength (*r* = 0.515, *p* = 0.017), and grip strength and peak inspiratory flow (*r* = 0.501, *p* = 0.021). During the study period, all subjects maintained good asthma control (Table 2) and good adherence to FFC pMDI and VFC Ellipta, with an average usage rate > 97%.

### Comparison of Overall Satisfaction Between Inhalation Devices

Data for the primary endpoint, i.e. the results of the Q5 questionnaire item, are shown in Fig. 2. Overall satisfaction was significantly higher with the FFC pMDI than with the VFC Ellipta in group 2 (*p* = 0.009), but not group 1 (*p* = 0.690), as shown in Fig. 2. On multivariate analysis, factors affecting overall satisfaction in total subjects were different by device (Ellipta vs. pMDI, odds ratio [OR] 2.86, *p* = 0.029), duration from onset of asthma (< 9.5 vs. ≥ 9.5 months, OR 3.57, *p* = 0.011), and previous medication (budesonide/formoterol Turbuhaler vs. fluticasone propionate/salmeterol xinafoate Diskus, OR 5.62, *p* = 0.012). Factors affecting overall satisfaction in group 2 differed by inhalation device (Ellipta vs. pMDI, OR 5.77, *p* = 0.024) and BMI (≥ 23.1 vs. < 23.1 kg/m^2^, OR 7.98, *p* = 0.010). However, other factors did not affect overall satisfaction with the FFC pMDI or the VFC Ellipta in group 2.

### Results of Inhaler Questionnaire (B) for the Degree of Satisfaction at Week 4

The results of the patient satisfaction questionnaire on inhalation devices are shown in Table 3. There were significantly more patients who felt bothered about the VFC Ellipta usage than the FFC pMDI usage in both groups 1 (*p* = 0.0238) and 2 (*p* = 0.0012), but this was more evident with group 2. Detailed reasons for negative answers to Q1 are shown in Table 3. The most frequent answers were the sensation of a powdery substance in the oral cavity and choking, shape of the mouthpiece, and discomfort of the mouth and tongue with the VFC Ellipta usage in both groups 1 and 2. Answers to Q3 and Q4 showed no significant differences between VFC Ellipta and FFC pMDI in either group 1 or 2. The average Q1, Q3, Q4, and Q5 scores were significantly higher with FFC pMDI usage in group 2 (*p* = 0.0168).

### Results of Evaluation Questionnaire (C) Concerning Device Operability at Week 4

The results of patients’ evaluations of device operability are shown in Table 5. Seventy percent or more of patients answered 4 (fair) or 5 (good) points for Q1 to Q4 with VFC Ellipta and FFC pMDI usage in both groups 1 and 2. Most patients with both the Ellipta and FFC pMDI devices answered that they were easy to understand, carry, prepare, and inhale. Concerning Q5, 12/22 (54.5%) and 14/21 (66.7%) patients did not feel that the smell bothered them with FFC pMDI usage in groups 1 and 2, respectively. Concerning Q6, 13/23 (56.5%) and 14/22 (63.6%) patients felt conscious of the medication while inhaling with the VFC Ellipta and FFC pMDI, respectively, in group 1. In group 2, 14/21 (66.7%) and 14/21 (66.7%) patients also felt conscious of the medication with the VFC Ellipta and FFC pMDI, respectively. Concerning Q7, 18/23 (78.3%) and 16/22 (72.7%) patients in group 1 answered that they could inhale correctly with the VFC Ellipta and FFC pMDI, respectively. In group 2, 15/21 (71.4%) and 14/21 (66.7%) patients answered that they could inhale correctly with the VFC Ellipta and FFC pMDI, respectively. Concerning Q8, 19/23 (82.6%) and 14/22 (63.6%) patients in group 1 answered 4 (fair) or 5 (good) points for the VFC Ellipta and FFC pMDI, respectively. In group 2, 13/21 (61.9%) and 16/21 (76.2%) patients answered 4 (fair) or 5 (good) points to Q8 regarding their overall impression with the VFC Ellipta and FFC pMDI, respectively. Considering the total results for Q1–Q8, device operability did not differ significantly between the FFC pMDI and VFC Ellipta in group 1 (*p* = 0.189) or 2 (*p* = 0.506).

### Device Handling Error Counts at Week 4

There were no device handling errors with the VFC Ellipta or FFC pMDI in group 1 at week 4 (Fig. 3). However, handling errors with both devices occurred at week 4 in group 2. The errors at week 4 in VFC Ellipta usage in group 2 included two cases of not opening the cover until they heard a clicking sound (no. 2 of the checklist), one case of blocking the air vent of the device with a finger while inhaling (no. 4 of the checklist), and six cases of no performance of strong and deep inhalation (no. 7 of the checklist). With FFC pMDI usage, one case of error timing consistency between inhalation and the medication spray (no. 9 of the checklist) was observed at week 4 in group 2. However, there were no significant differences in device operability errors between the FFC pMDI and VFC Ellipta in group 2 (*p* = 0.506).

### Respiratory Resistance

The results for respiratory resistance are shown in Table 6. The X5 and ALX items changed significantly during FFC pMDI usage in group 1. There were significant differences in X5, Fres, and ALX between the VFC Ellipta and FFC pMDI in group 1. However, there were no significant changes over the study in group 2.

**Table 6:** Results of respiratory function [respiratory impedance measured by the forced oscillation technique (FOT)]

	Group 1 (DPI:23 pMDI:22)					Group 2 (DPI:21 pMDI:21)				
	Week 0	Week 4	Within-group change	Within-group *p* value*	Between-group *p* value**	Week 0	Week 4	Within-group change	Within-group *p* value*	Between-group *p* value**
R5 (cmH_2_O/L/s)										
VFC	2.37	2.57	0.20	0.311	0.913	2.86	3.07	0.21	0.448	0.792
FFC	2.34	2.53	0.07	0.318		3.08	3.22	0.15	0.284	
R20 (cmH_2_O/L/s)										
VFC	2.30	2.44	0.06	0.575	0.939	2.69	2.82	0.13	0.511	0.831
FFC	2.38	2.42	0.02	0.342		2.78	2.83	0.05	0.687	
R5–R20 (cmH_2_O/L/s)										
VFC	−0.01	0.13	0.14	0.290	0.809	0.17	0.25	0.08	0.300	0.554
FFC	0.05	0.11	0.06	0.439		0.29	0.39	0.10	0.219	
X5 (cmH_2_O/L/s)										
VFC	−0.30	−0.30	−0.01	0.883	0.020	−0.53	−0.55	−0.02	0.602	0.970
FFC	−0.20	−0.41	−0.18	0.001		−0.52	−0.58	−0.06	0.545	
Fres (Hz)										
VFC	8.15	7.72	−0.43	0.148	0.040	10.23	10.34	0.11	0.468	0.302
FFC	7.47	8.64	0.84	0.053		10.55	10.05	−0.50	0.448	
ALX (cmH_2_O/L/s Hz)										
VFC	0.95	1.39	0.001	0.250	0.006	2.82	3.06	0.23	0.556	0.744
FFC	1.39	1.84	0.81	0.002		2.84	2.99	0.15	0.828	

*R5* respiratory resistance at 5 Hz, *R20* respiratory resistance at 5 Hz, *X5* low-frequency reactance indices at 5 Hz, *Fres* resonant frequency, *ALX* low-frequency reactance area

*Within-group differences were analysed by the Wilcoxon signed-rank test

**Between-group differences were analysed by the Wilcoxon rank-sum test

### Adverse Events

One serious adverse event, acute appendicitis, which required inpatient hospitalization, occurred in group 2. Other non-serious adverse events were as follows: cough in three cases, hoarseness in two, eczema in two, and one event each of induction of cough, osteoarthritis, acute bronchitis, acute upper respiratory inflammation, altered taste, infectious gastroenteritis, and incontinence.

## Discussion

In this study, patient satisfaction and preference with regard to two different inhaled medications, VFC Ellipta and FFC pMDI, were compared in a ≤ 64-year-old group (group 1) and a ≥ 65-year-old group (group 2) after three preconditions: stable asthma control (Table 2), drug adherence rate ≥ 97%, and appropriate inhalation technique. No significant difference in patient satisfaction was seen between the two inhaled medications in group 1 (*p* = 0.690), but in group 2, patient satisfaction was found to be significantly higher with the FFC pMDI than with the VFC Ellipta (*p* = 0.009) (Fig. 2). Two factors affected the significantly higher level of satisfaction with the FFC pMDI in group 2: differences in the devices and BMI. Of the differences in the devices, the FFC pMDI device was associated with significantly greater patient satisfaction than the Ellipta device (OR 5.77, *p* = 0.024). However, the results of the questionnaire on device operability for VFC Ellipta and FFC pMDI showed no significant differences in any of the individual questions Q1–Q8, and no significant difference in an assessment of Q1–Q8 overall (*p* = 0.506) (Table 5). Why, then, does the pMDI device have a significantly higher level of satisfaction among elderly people?

First, subjects responded that the FFC pMDI was significantly less bothersome than the VFC Ellipta, as shown in the results for Q1 (Table 3). The responses to Q2 on the specific things that were felt to be bothersome are shown in Table 4. In group 2, specific examples of difficulties with the VFC Ellipta that were reported can be roughly categorized into two types: those related to the formulation physical properties, such as a “powdery feeling” and “discomfort in the mouth and tongue,” and those related to the device, such as the mouthpiece and lever. More than half of the subjects in group 2 had some kind of complaint. In contrast, specific examples of the negative reactions to the FFC pMDI were mainly two cases of difficulty in coordinating breathing and two cases regarding the smell of the drug; the absolute number of complaints itself was smaller than with the VFC Ellipta. The negative reaction as a result of the difficulty in coordinating breathing with this pMDI preparation is something that was already known before the introduction of the drug, deriving from device characteristics of the pMDI preparation [13], and is not something new in this study. The use of a spacer is an effective way to counter this, although spacers were not used in this study. In previous reports that used conventional pMDI preparations, while there were differences in the degree of negative response depending on age cohort and other factors, 24.4–81.4% of patients were reported to have difficulty in coordinating their breathing [9, 10, 14]. In the present group 2, however, only two subjects reported difficulty in coordinating breathing as a specific reason that this device was bothersome. This was less than 10% of group 2 overall, and much less than in previous reports [15]. One point of difference between the FFC pMDI and conventional pMDI is that the FFC pMDI was developed so that the spray velocity of the drug would be slower, to make breathing coordination easier, and a spacer is usually needed [16]. It is speculated that one of the reasons for the significantly higher satisfaction with the FFC pMDI in group 2 may have stemmed from this feature of the FFC pMDI device.

The level of satisfaction with the pMDI in group 2 was also found to be significantly higher in patients with lower BMI (OR 7.98, *p* = 0.010). Why was the level of satisfaction with pMDI significantly higher in patients with lower BMI? As shown in Table 1, despite no significant difference in BMI between groups 1 and 2 (*p* = 0.659), the patients in group 2 had significantly lower grip strength, finger muscle strength, and peak inspiratory flow than those in group 1 (all *p* < 0.05). These data may be taken as a characteristic of group 2 as a group of elderly patients. Looking at the correlations of these parameters with BMI in group 2, a weak correlation was seen between BMI and grip strength (*r* = 0.374, *p* = 0.095), and relatively strong correlations were seen between grip strength and finger muscle strength and between grip strength and peak inspiratory flow (*r* = 0.515, *p* = 0.017 and *r* = 0.501, *p* = 0.021, respectively). While these data express different things, they may be taken as showing different aspects of decreased muscle strength in elderly people.

With the Ellipta device, the action of opening the cover loads the drug and prepares the blister for inhalation of the medication, a feature of which is a squeaking sound together with fairly strong resistance. This requires a certain amount of finger muscle and grip strength, and while it had little effect in group 1 patients, who had sufficient strength (Table 1), it may have been felt to be a burden by patients in group 2, who had significantly less grip strength. Moreover, since the VFC Ellipta is a DPI preparation, the dry powder needs to be strongly and deeply inhaled at the time of inhalation, which demands a sufficient peak inspiratory flow level in patients. Therefore, the drug cannot be satisfactorily inhaled in some cases when patients do not have the muscle strength needed to inhale strongly, such as in the respiratory muscles and diaphragm. On the other hand, since the FFC pMDI has a relatively stiff cylinder, this study was conducted with all patients using an inhalation assistance device (Full Push) from the beginning. Therefore, among elderly patients with low BMI, the device could be used readily despite significantly decreased grip and finger muscle strength, and this was thought to be reflected in the results showing significantly higher levels of satisfaction compared with the VFC Ellipta. This is consistent with the finding that in group 2, there tended to be a better response for the FFC pMDI to Q4 in Table 3.

The process by which patients use the device and inhale the medication was broadly divided into two components: the process of setting the drug in the device to prepare for inhalation (a device operability aspect) and the process of actual inhalation by the patients (a drug inhalation aspect). Modifications and improvement in the former can be expected with patient education by repeatedly going through the appropriate operation method and proper procedures. In this study, the subjects were fully instructed on inhalation before they started to use each device, and no significant differences were seen in any of Q1–Q8 concerning the evaluation of device operability at week 4, nor were any significant differences seen in the evaluation of the overall impression of the device in Q1–Q8 (*p* = 0.506).

For the latter, however, much was attributable to the patients’ own physical ability, and though improvement could be expected from training in younger people, in many cases there was a cut-off age beyond which improvement could not be anticipated in elderly people [17, 18]. For peak inspiratory flow and grip and finger muscle strength, there are limitations to what elderly patients can do themselves. The BMI of the study subjects was around 23 kg/m^2^, which is the Japanese average. No patient was obese, and so it may be supposed that body weight reflected muscle mass, and that when BMI was low, muscle mass was also low. This is consistent with low grip strength and finger muscle strength. The reason for the significantly higher satisfaction with the FFC pMDI than the VFC Ellipta in the elderly patient group was thus not only the type of device used; the fact that BMI had a significant effect is useful information when selecting the device for elderly people. Another major advantage to using the pMDI is that the adverse circumstances affecting inhaler coordination of individual patients can be dealt with by using options such as spacers or valved holding chambers as auxiliary devices [19]. Especially in elderly people with decreased muscle strength and physical ability, it is possible that a feeling of achievement can be obtained from effective inhalation with the use of auxiliary devices, leading to higher levels of satisfaction and preference.

A point to be noted is that, despite full guidance and confirmation of the appropriate inhalation technique before the study, mistakes in operation of both devices appeared after 4 weeks in group 2 (Fig. 3). With the VFC Ellipta, errors included that the cover was not fully opened, a finger blocked the air hole, and the patient did not inhale strongly and deeply during inhalation, whereas with the FFC pMDI, a case occurred in which the patient could not coordinate breathing when the drug was sprayed. In a report comparing conventional pMDI and DPI preparations Diskus, HandiHaler, and Turbuhaler, operation errors were significantly higher with these DPI preparations than with the pMDI in an elderly patient group, despite inhalation instruction [20]. In a different study, operation errors were also significantly higher with prefilled dry power inhaler DPI preparations [21]. In a reported investigation of patients with asthma and those with chronic obstructive pulmonary disease, 59% of patients made operation errors at least once after receiving inhalation instructions. Mistakes were seen in the inhalation operation during breathing coordination with the conventional pMDI and in operation of the device with DPI. All were related to low air intake speed [22].

Many patients in group 2 gave fair (4 points) or good (5 points) responses to questions Q1–Q8 (Table 5), and there were no significant differences between the Ellipta and pMDI. In group 2, the operation errors that occurred after 4 weeks cannot be expressed with the response to questions on this questionnaire because most patients reported “fair” or “good” about device operability, and it is possible that, though the patients themselves thought that they were using the devices properly, operation errors occurred unconsciously. This suggests that inhalation guidance needs to be repeated.

The types of patient adherence, both for the drug and for the inhalation device, need to be simultaneously stored in accordance with the instructions for use and operated correctly for the effective continuation of inhalation therapy. This requires that physicians, pharmacists, and other healthcare professionals provide not only instruction for taking the medication, but also repeated instruction in the inhalation technique. This helps ensure that the inhalation device is always used with an appropriate operational technique and procedure so that effective inhalation therapy can continue. One study showed that the feeling that the drug could be effectively inhaled produces a sense of achievement in patients with regard to the therapy, leading to satisfaction and preference toward the treatment [23]. Such patient satisfaction and preference are important feedback as a key to maintaining good adherence, leading to the continuation of effective inhalation therapy. The results obtained in this study showed significantly higher patient satisfaction with the FFC pMDI in older patients, but there were differences in satisfaction and preferences among patients, which may change with time based on various factors, such as age. In clinical settings, there are frequent switches from the VFC Ellipta to the FFC pMDI or vice versa. The results obtained in this study may provide useful hints at such times.

## Conclusion

FFC pMDI showed significantly higher satisfaction and preference levels in elderly stable patients than VFC Ellipta when asthma control level, adherence, and device operability were well maintained during the study period.

## Supplementary Information

Below is the link to the electronic supplementary material.

Supplementary file1 (PDF 22 kb)

Supplementary file2 (PDF 87 kb)
