# Supplementary material for: Comparison of User Satisfaction and Preference with Inhalant Devices Between a Pressurized Metered-Dose Inhaler and Ellipta in Stable Asthma Patients: A Randomized, Crossover Study
Source: Pulm Ther. 2021 Mar 2;7(1):171–87. doi: 10.1007/s41030-021-00149-6 (PMC8137762; doi:10.1007/s41030-021-00149-6)
Supplement: Supplementary file 1 — Supplementary file1 (PDF 22 kb) [file 41030_2021_149_MOESM1_ESM.pdf]

Supplement 1

Eligibility criteria

1) Adult outpatients with mild or moderate stable asthma (GINA treatment step 2 or 3)
2) Patients who provided written, informed consent after being given and understanding a detailed explanation of the study
3) Patients with asthma control questionnaire scores less than 0.75
4) Patients who have been using medium dose of dry powder type inhaled corticosteroid and long-acting beta 2 agonist (ICS/LABA) for treatment for more than 3 months before enrolment.
5) No histories of formoterol/fluticasone combination (FFC) as pMDI devise use and vilanterol/fluticasone combination (VFC) as Ellipta device use.
6) Patients who retained a good level of drug adherence to prior drug therapy after checking inhalation technique
7) Patients who could inhale adequately every time with no medical assistance.
8) Patients who accepted inhaling FFC pMDI without using an inhalation spacer.
