# Supplementary material for: Comparison of User Satisfaction and Preference with Inhalant Devices Between a Pressurized Metered-Dose Inhaler and Ellipta in Stable Asthma Patients: A Randomized, Crossover Study
Source: Pulm Ther. 2021 Mar 2;7(1):171–87. doi: 10.1007/s41030-021-00149-6 (PMC8137762; doi:10.1007/s41030-021-00149-6)
Supplement: Supplementary file 2 — Supplementary file2 (PDF 87 kb) [file 41030_2021_149_MOESM2_ESM.pdf]

Supplement 2

Questionnaire forms

(A) Questionnaire regarding the degree of patients' satisfaction at week 4

Patients answered the five statements below concerning each inhaler. Patients answered each questionnaire (except Q2) using the 10-cm visual analogue scale (VAS) form.

Question 1 (Q1). Did anything about the inhaler bother you? Answer: Yes, something bothered me a lot (VAS scale 0 cm). – No, nothing about it bothered me (VAS scale 10 cm).

Q2. If something about it bothered you, tell us about it.

Q3. Was the inhaler easy to use? Answer: It was very difficult to use (VAS scale 0 cm). – It was very easy to use (VAS scale 10 cm).

Q4. Do you want to continue using it? Answer: I have no desire to continue using it (VAS scale 0 cm). – I am eager to continue using it (VAS scale 10 cm).

Q5. Overall degree of satisfaction. Answer: Unsatisfied (VAS scale 0 cm) – Satisfied (VAS scale 10 cm).

(B) Questionnaire concerning the evaluation of device operability at week 4

Patients checked the box beside the statement regarding the evaluation of the inhaler that they agreed with.

There were five categories of answer checkboxes from bad to good with numbering from 1 to 5, as follows: Bad: 1 point, Poor: 2 points, Neutral: 3 points, Fair: 4 points, and Good: 5 points.

Q1. Are the usage instructions easy to understand?

Q2. Is the inhaler easy to carry?

Q3. Are the preparations for inhaling simple?

Q4. Is it easy to inhale?

Q5. Does the smell bother you?

Q6. Are you conscious of the medication while inhaling?

Q7. Do you feel that you are inhaling correctly?

Q8. What is your overall impression of the inhaler?

Inhaling guidance checklist

Patients were asked about device operability using the checklists below at weeks 0 and 4.

(A) FFC pMDI inhalation checklist

Before inhaling

Checkbox-

No.1: Before using inhalation drug for the first time or when using it after a long time, I perform a test spray.

No.2: I do not perform a test spray before daily use.

No.3: I gently shake the container of inhalation drug before inhaling.

No.4: I can hold the inhaler firmly with the correct fingers.

No.5: Before inhaling, I check the remaining medication indicator.

No.6: I exhale before inhaling, but only till I do not feel uncomfortable.

While inhaling

No.7: My finger presses the bottom of the container with effective force.

No.8: While inhaling, I face directly forward or slightly downwards.

No.9: I inhale and spray the medication at the same time.

No.10: The medication sprays in a constant direction.

No.11: I gently and deeply inhale the medication.

No.12: Immediately after inhaling, I close my mouth and hold my breath for almost 5 seconds.

After inhaling

No.13: After inhaling, I thoroughly rinse my mouth.

No.14: I replace the cap to keep the inhaler clean.

(B) VFC Ellipta inhalation checklist

Before inhaling

Checkbox No.1: Before inhaling, I check the remaining medication indicator.

No.2: I open the cover till I hear a clicking sound.

No.3: I exhale before inhaling, but only till I do not feel uncomfortable.

While inhaling

No.4: I do not block the air vent with my finger while inhaling.

No.5: Just before inhaling, I do not breathe on the mouthpiece.

No.6: I do not start inhaling until my mouth is on the mouthpiece.

No.7: I inhale strongly and deeply.

No.8: I hold the inhaler level as I inhale.

No.9: While inhaling I keep the corners of my mouth closed.

No.10: I do not breathe in and out with my mouth on the mouthpiece.

No.11: While inhaling, I do not block the mouthpiece with my tongue.

No.12: Immediately after inhaling, I close my mouth and hold my breath for almost 5 seconds.

After inhaling

No.13: After inhaling, I firmly close the cover.

No.14: After inhaling, I thoroughly rinse my mouth.
